# Identification of Plasma Lipidome Changes Associated with Low Dose Space-Type Radiation Exposure in a Murine Model

**DOI:** 10.3390/metabo10060252

**Published:** 2020-06-17

**Authors:** Maarisha Upadhyay, Meena Rajagopal, Kirandeep Gill, Yaoxiang Li, Shivani Bansal, Vijayalakshmi Sridharan, John B. Tyburski, Marjan Boerma, Amrita K. Cheema

**Affiliations:** 1 Department of Oncology, Lombardi Comprehensive Cancer Center, Georgetown University Medical Center, Washington, DC 20057, USA; maarisha.u@gmail.com (M.U.); meena.rajagopal@nih.gov (M.R.); kkg30@georgetown.edu (K.G.); yl814@georgetown.edu (Y.L.); sm3451@georgetown.edu (S.B.); tyburskijb@gmail.com (J.B.T.); 2 Division of Radiation Health, University of Arkansas for Medical Sciences, 4301 West Markham Slot 522-10, Little Rock, AR 72205, USA; vmohanseenivasan@uams.edu (V.S.); MBoerma@uams.edu (M.B.); 3 Department of Biochemistry, Molecular and Cellular Biology, Georgetown University Medical Center, Washington, DC 20057, USA

**Keywords:** lipidomics, oxygen radiation, plasma metabolomics, ionizing radiation, untargeted profiling, mass spectrometry

## Abstract

Long-term exposures to low dose space radiation may have adverse effects on human health during missions in deep space. Conventional dosimetry, monitoring of prodromal symptoms, and peripheral lymphocyte counts are of limited value as biomarkers of organ- and tissue-specific radiation injury, particularly of injuries that appear weeks or months after radiation exposure. To assess the feasibility of using plasma metabolic and lipidomic profiles as biomarkers of injury from space radiation, we used a mouse model of exposure to low doses of oxygen ions (^16^O) and protons (^1^H). Plasma profiles were compared with those of mice exposed to γ-rays as a reference set. Our results demonstrate major changes in glycerophospholipid metabolism, amino acid metabolism, as well as fatty acid metabolism. We also observed dyslipidemia and lipid peroxidation, suggesting an inflammatory phenotype with possible long-term consequences to overall health upon exposure to low doses of high linear energy transfer (LET) radiation.

## 1. Introduction

Crew members engaged in space exploration missions run a finite risk of exposure to ionizing radiation (IR) including galactic cosmic rays (GCR), and radiation from solar particle events. Despite improvements in radiation shielding technology and advances in biomedical countermeasures to protect astronauts from the damaging effects of space radiation, exposure to radiation still remains a threat to astronaut health during National Aeronautics and Space Administration’s (NASA) deep space missions including planned expeditions to Mars [1,2]. The GCR spectrum consists of nearly 87% protons (^1^H), 12% helium ions and the remaining ~1–2% consists of high charge and energy (HZE) nuclei that include oxygen ions (^16^O) [3]. Hence, when travelling outside of the Earth’s orbit, the crewmembers run a risk of continuous exposure to low doses of protons and heavier ions like ^16^O [4]. Chronic radiation exposure from GCR occurs at a dose rate of 1.3 milligray per day (mGy/day) while crewmembers returning from a mission to Mars can be exposed to cumulative dose of 0.5 Gy [4,5,6]. Due to its higher relative biological effectiveness (RBE) compared to and proton radiation [7,8], heavy ion radiation is expected to have greater potential to cause macromolecular damage including DNA damage and lipid peroxidation events.

Radiation is known to promote oxidative stress and inflammation through alterations in biochemical pathways leading to acute effects such as cell death and gastrointestinal (GI) mucositis as well as chronic effects such as persistent inflammation, cellular transformation, and cancer [9]. However, the extent of macromolecular damage depends on the radiation type, total dose and dose rate. Several studies have contributed to the understanding of radiation induced molecular damage and tissue injury [10,11], however, there is no consensus about the health risks of low dose high- linear energy transfer (LET) radiation. Prior studies have reported oxidative stress in the central nervous system of mice due to exposure to low-LET radiation at doses <1 Gy in chronic low dose rates [12]. Other laboratories have shown behavioral deficiencies and increased cardiovascular risk associated with low dose ^1^H and HZE radiation [13,14]. Though the majority of reports on health effects of γ-radiation is in the context of medical exposure, metabolic alterations that are dose dependent and across various time points have also been reported [15,16]. As such, there is a need to tease out molecular changes indicative of radiation exposure from those that can help predict radiation-induced tissue injury. 

**Figure 1:**
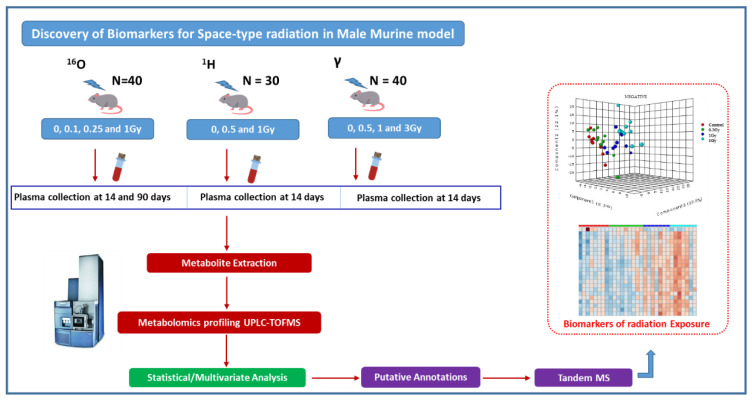
Workflow of plasma metabolomics using male C57BL/6J mice exposed to sham or low doses of space type radiation.

Previously, we have shown that exposure to heavy ions (^56^Fe) cause oxidative stress and dysregulated prostanoid biosynthesis in the mouse intestinal metabolome [17]. In this study, we sought to understand alterations in plasma metabolome in mice exposed to low doses of space type radiation. For this purpose, separate cohorts of adult male C57BL/6J mice were exposed to 0.5 Gy and 1 Gy of ^1^H (150 MeV) with plasma collection at 14 days’ post-irradiation, or to 0.1 Gy, 0.25 Gy and 1 Gy of ^16^O (600 MeV/n) with plasma collection at days 14 and 90 (Figure 1). Another cohort of mice was exposed to 0.5 Gy, 1 Gy or 3 Gy of γ-rays as a reference set for low-LET radiation with plasma collection at 14 days’ post-irradiation. We report the findings from metabolomic and lipidomic profiling studies that identified radiation type, and dose- and time-dependent metabolic alterations. Interestingly, mice irradiated with 0.1 Gy of ^16^O radiation showed maximal alterations in metabolomic and lipidomic profiles monitored over a 90-day time frame, indicating a hyper sensitive response to low doses of high-LET radiation. Our findings demonstrate that exposure to high-LET ^16^O radiation led to distinct alterations in plasma level metabolic profiles that are suggestive of an inflammatory phenotype.

## 2. Results

In this study, male C57BL/6J mice were exposed to low doses of high-LET radiation that are mission-relevant to NASA. A separate cohort of mice was exposed to γ-rays as reference radiation. We performed untargeted metabolomics and lipidomics analyses of plasma samples collected at 14 and 90 days after irradiation to identify altered metabolites using high resolution mass spectrometry in conjunction with ultra-performance liquid chromatography (UPLC-QToF MS). Plasma profiles within each radiation group were compared using ANOVA to identify features that were dysregulated over time for each radiation type. Student’s *t*-tests were also performed between controls and irradiated plasma profiles, for each of the time points for a given radiation group. 

### 2.1. Exposure to ^16^O Radiation Elicits Robust Changes in Plasma Lipidome 

**Figure 2:**
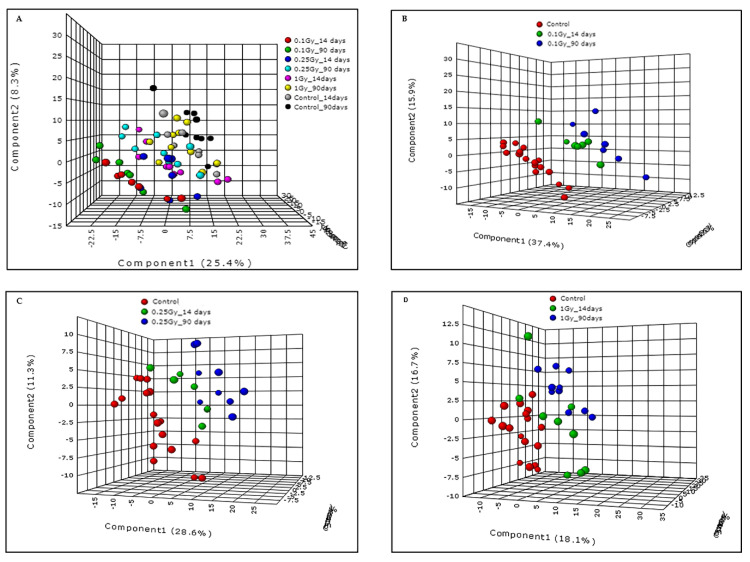
Exposure to low doses of ^16^O radiation causes alterations in plasma molecular profiles. Panel (**A**): PLS-DA demonstrating separation between irradiated and sham groups across all doses and time points in this study. Panel (**B**): PLS-DA analyses showing clear separation between 0.1 Gy radiation group and sham at both 14 and 90 days post-irradiation. Panels (**C** and **D**): PLS-DA plots showing group separation for mice exposed to 0.25 Gy and 1 Gy as compared to sham.

We performed metabolomic/lipidomic profiling of plasma samples obtained from sham mice (*n* = 9) and those exposed to 0.1 Gy (*n* = 7), 0.25 Gy (*n* = 9) or 1 Gy (*n* = 10) of ^16^O (600 MeV/n) radiation. Pre-processing of liquid chromatography (LC)-mass spectrometry (MS) data was performed using XCMS peak picking software (Scripps Institute, La Jolla, CA, USA) and resulted in 2639 and 1989 features in electrospray positive and negative modes, respectively. The pre-processing was followed up by quality control measures which involve calculation of the normalized intensities based relative standard deviation (RSD) for each feature. The features with more than 15% of coefficient of variation (CV) were filtered out, resulting in the removal of 356 and 233 features in electrospray positive and negative modes, respectively. Initially, we performed principal component analysis that did not result in a clear group separation (data not shown). Hence, a partial least squares discriminate analysis (PLS-DA) model (Metaboanalyst v3.0, Alberta, Canada) was performed to examine group separation between mice exposed to varying doses of ^16^O or sham irradiation (Figure 2, panel A). The R^2^ and Q^2^ for the OPLS (orthogonal partial least square) model were 0.9 and 0.65 respectively in the negative mode, supporting the quality of the model.

**Figure 3:**
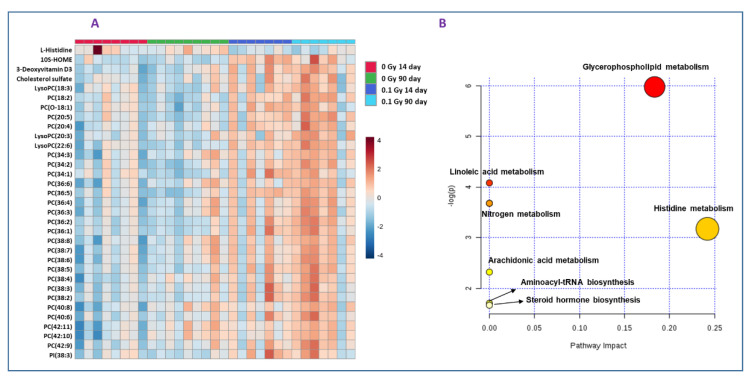
Dysregulated metabolites and biochemical pathways of low dose high-LET radiation. Panel (**A**): Hierarchical clustering analysis of differential metabolite expression in the irradiated and sham groups. Panels (**B**): Pathway analysis of differentially abundant metabolites identified in plasma from mice exposed to 0.1 Gy ^16^O compared to sham using MetaboAnalyst 3.0.

While the PLS-DA plot indicates separation between all groups compared (Figure 2, panel A), maximal separation was observed for mice that received 0.1 Gy of ^16^O radiation. The metabolic changes were highly significant in plasma collected 90 days’ post-irradiation, indicating that the metabolic and lipidomic alterations were stable over time (Figure 2, Panel B). However, as shown in Figure 2, panels C and D, the inter-group variability decreased as a function of dose (0.25 Gy or 1 Gy). Subsequently, hierarchical clustering analysis was performed to visualize the differential expression of metabolites between the control and irradiated groups (Figure 3, panel A). Out of 175 metabolites that were found to be dysregulated in the plasma of mice exposed to 0.1 Gy of ^16^O, the identities of 51 metabolites and lipids were confirmed by matching MS/MS (Tandem-Mass Spectrometry) fragmentation spectra for each analyte against the NIST (National Institute of Standards and Technology) or METLIN (Scripps Institute, La Jolla, CA, USA) databases; this pipeline has been used by several research groups [18,19,20] (Supplementary Table S1). 

Exposure to 0.1 Gy of ^16^O at 14 days and 90 days resulted in dysregulation of a large number of metabolites and lipids; however, a majority of these features could not be annotated using tandem mass spectrometry which remains a major bottleneck in this area of research. Using MS/MS fragmentation matching with the NIST METLIN databases, we were able to identify several glycerophospholipids including free fatty acids, phosphatidylcholines (PCs) and lyso phosphatidylcholines (LPCs) that were significantly higher in plasma of 0.1 Gy-irradiated mice compared to sham mice. The fragment information is detailed in Supplementary Table S1.

Further, we performed pathway enrichment analyses using Metaboanalyst v3.0 software for the dysregulated metabolites observed in mice plasma exposed to 0.1 Gy ^16^O radiation. Several pathways including glycerophospholipid, fatty acids metabolism such as linoleic acid and arachidonic acid metabolism, steroid hormone biosynthesis and histidine metabolism were found to be significantly perturbed (Figure 3B). 

**Figure 4:**
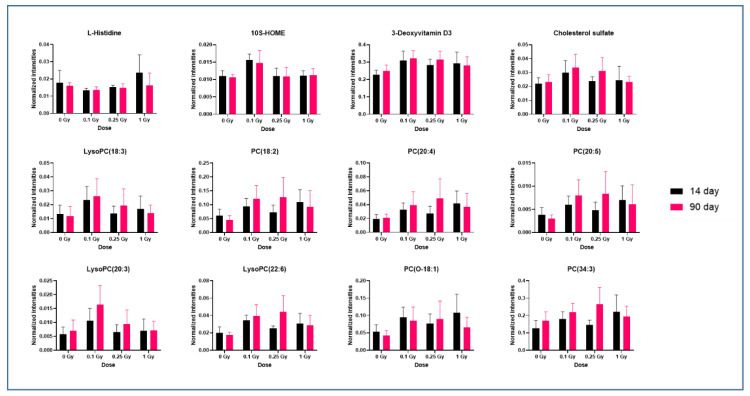
Exposure to low dose high-LET radiation causes dyslipidemia. Box plots for relative response of representative metabolites in mice exposed to sham, 0.1 Gy, 0.25 Gy or 1 Gy of ^16^O radiation at days 14 and 90 post-irradiation.

Next, we examined time- and dose-dependent response of a panel of altered metabolites to ^16^O radiation using box plots (Figure 4); the dysregulated metabolites for each comparison are listed in Supplementary Tables S2 and S3, respectively. Details of one-way ANOVA and Tukey’s HSD post-hoc analyses information for these plots is included in Supplementary Table S4. Lipids were the major class of dysregulated metabolites that higher abundance in plasma of irradiated mice at 14 days and 90 days. Significantly upregulated lipids included PC(20:4), PC(18:2), PC(20:5), PC(38:5) and LysoPC(18:3), LysoPC(20:3) and LysoPC(22:6) at 14 days after exposure to 0.1 Gy ^16^O.

On the other hand, plasma levels of amino acid histidine decreased at 0.1 Gy exposure; however, the levels rose to near normal with increase in radiation dose and time of exposure. We also observed significant changes in deoxyvitamin D3, cholesterol sulfate and octadecenoic acid (HOME). Dysregulation of aldosterone was observed at 1.0 Gy of ^16^O radiation when compared to the sham group. 

### 2.2. Metabolic Changes in Mouse Plasma Following Exposure to ^1^H

**Figure 5:**
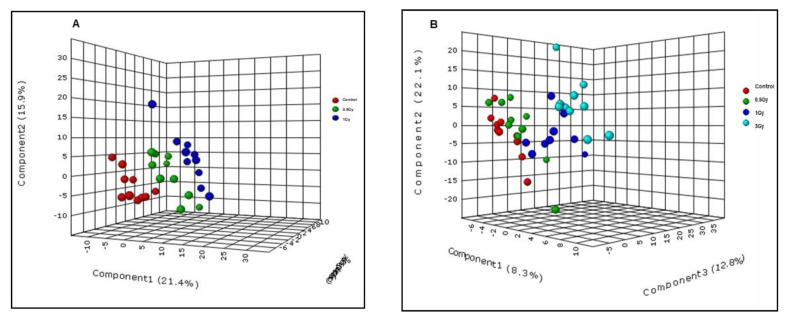
Alterations in mouse plasma metabolite profiles in response to ^1^H and γ-irradiation. Panel (**A**): PLS-DA plot showing ANOVA comparison of samples from 0.5 Gy and 1 Gy exposed mice versus control (sham) in response to ^1^H irradiation in the negative ionization mode. Panel (**B**): PLS-DA plot showing ANOVA comparison of 0.5 Gy, 1 Gy and 3 Gy-irradiated samples versus control (sham) after exposure to γ-irradiation in the negative ionization mode.

We exposed male C57BL/6J mice to 0.5 Gy (*n* = 10) and 1 Gy (*n* = 10) of ^1^H (150 MeV) radiation and performed high resolution mass spectrometry-based metabolomics/lipidomics with plasma samples collected at 14 days’ post-irradiation. There was a clear separation of groups as depicted by the PLS-DA score plot (Figure 5, panel A). Moreover, binary comparison revealed several metabolites that were significantly altered after exposure to 1 Gy ^1^H irradiation versus sham. The levels of uridine and glycerophospholipid were significantly higher in plasma of 1 Gy ^1^H-irradiated mice (Supplementary Table S5) relative to sham samples. 

### 2.3. Low LET γ-Radiation Induced Changes in Plasma of Male C57BL/6J Mice 

We examined the metabolic/lipidomic perturbations in plasma of mice irradiated with 0.5 Gy (*n* = 10), 1 Gy (*n* = 10) and 3 Gy (*n* = 10) of γ-radiation or sham at 14 days after exposure (LD_50_ ~ 8.8Gy [21,22]). These doses of γ-radiation were chosen as reference plasma profiles for comparative low-LET radiation response. UPLC-QToF MS analysis of plasma revealed approximately 2639 and 1989 features in the positive and negative ionization modes, respectively. PLS-DA analysis showed clear separation between sham and the 3 Gy γ-irradiated group, indicating inherent differences in the metabolic profiles of two cohorts of mice (Figure 5, panel B). While the profiles of the 0.5 Gy cohort were closer to sham, the 1 Gy-irradiation group showed intermediate separation and the 3 Gy-irradiation group showed maximum separation from the sham group. ANOVA analyses helped to delineate several dysregulated metabolites of which seven were verified using tandem mass spectrometry as significantly altered due to γ-irradiation. These included glycerophosphocholine, phosphatidic acid, N-oleoyl histidine and amino acid methionine, which were in accordance with previously published reports [21,23,24,25]. Moreover, among the radiation types studied here, the alteration in methionine levels was observed only in the γ-irradiation exposure group. A combined list of dysregulated metabolites for ^16^O and ^1^H radiation groups are listed in Supplementary Table S6.

## 3. Discussion 

Future deep space missions are likely to impose health risks to crew members due to potential exposure to space radiation. Delineating molecular changes in plasma are likely to further our understanding of systemic alterations with long-term health consequences. Given the higher relative biological effectiveness compared to γ-rays, charged-particle high-LET radiation is expected to cause metabolic dysregulation even at low doses [23,26,27]. Most previous studies assessing the effects of high-LET radiation have focused on high doses and shown detrimental health effects ranging from the acute radiation syndrome to late effects of radiation. However, there is little understanding of molecular changes that may accompany low dose high-LET radiation. In this study, we sought to elucidate metabolic and lipidomic changes in plasma of adult male C57BL/6J mice exposed to low doses of high-LET radiation. A total of 110 mice were exposed to ^16^O, protons or γ-rays (as a low-LET reference group). We analyzed 101 plasma samples collected from these mice at 14 days after exposure, using high resolution mass spectrometric analyses. To study the long-term effects of exposure to ^16^O radiation, the total of 35 samples were also collected at 90 days’ time point. 

We observed that exposure to 0.1 Gy ^16^O radiation caused robust changes in metabolomic and lipidomic profiles at the 14-day time point; some of these were stable over 90-day time period. However, plasma profiles of animals irradiated with 0.25 or 1 Gy were found to be close to the control group. Plasma levels of lipids such as PC(20:4), PC(18:2), PC(20:5), PC(38:5), LysoPC(18:3), LysoPC(20:3) and LysoPC(22:6) were significantly (+ 2 fold, *p* value ≤ 0.05) increased at 14 days after exposure to 0.1 Gy ^16^O. These results are in agreement with previous reports in which ^16^O exhibited a higher degree of change in lipids compared to ^1^H [28,29]. An increase in overall plasma PC levels with a concomitant rise in LysoPCs levels is suggestive of oxidative stress and inflammation upon exposure to ^16^O [30]. Alterations in metabolome following exposure to low dose high-LET radiation have previously been reported by our group [31]. Apart from being the major constituents of cellular membrane bilayer, PCs also play important roles in cell-signaling through the generation of LPCs, phosphatidic acid and diacylglycerols. Reactive oxygen species (ROS), often present at higher levels in radiated-exposed animals, might lead to upregulation of PCs and LPCs [32]. Additionally, free radicals could also cause membrane lipid peroxidation resulting in increased lipids in the circulation [33]. Radiation exposure is known to induce lipid peroxidation and dyslipidemia ultimately leading to cellular damage [34,35]. 

Dysregulation of arachidonic acid metabolism can lead to radiation-induced tissue injury by inducing tissue fibrosis [36]. We also noticed a significant dysregulation in oleic acid and palmitic acid levels. Apart from being important constituents of membrane matrix, fatty acids also serve as secondary messengers [37]. Radiation-induced disruption of amino acid metabolism is well documented [38,39]. We observed significant dysregulation in levels of L-histidine, an anti-inflammatory metabolite [39], upon exposure to 0.1 Gy of ^16^O radiation. This could promote an inflammatory tissue environment after ^16^O radiation. On the other hand, increased levels of L-histidine were seen in plasma of mice exposed to 1 Gy ^16^O. This could be attributed to increased protein degradation and/or catabolism.

We also observed alterations in plasma levels of glycyl-serine, asparaginyl-cysteine and theroninyl-aspartate dipeptides after ^16^O irradiation, which may indicate alteration in either the dipeptidase activity or dipeptide transport across intestinal epithelial cells. Additionally, aldosterone dysregulation observed at 1.0 Gy of ^16^O radiation indicates perturbation in steroid hormone biosynthesis pathways. Aldosterone plays an important pathophysiological role in hypertension, atherosclerosis, heart failure, myocardial infarction and cardiac hypertrophy [40]. Dysregulations in cholesterol levels are associated with increased risk of cardiovascular diseases [22,25,41] and cancer [42,43]. 

Exposure to 1 Gy of ^1^H radiation caused modest changes in plasma metabolite profiles. Nonetheless, an upregulation in uridine in plasma after 1 Gy of ^1^H suggests perturbed nucleotide metabolism possibly resulting from macromolecular damage upon irradiation [7,44,45]. Other dysregulated metabolites included prostaglandin-EA, N-arachidonoyl tyrosine and indole. We also observed transient alterations in plasma levels of prostaglandin-H2 and N-acyl amino acids. These results are in agreement with a study by Chang et al. who reported that 0.5 Gy is the threshold dose of ^1^H (150 MeV) to cause metabolite changes in mice. 

## 4. Materials and Methods 

### 4.1. Animals and Radiation Exposure

Male C57BL/6J mice (1–2 months old) were purchased from the Jackson Laboratory (Bar Harbor, ME, USA) and delivered to the University of Arkansas for Medical Sciences (UAMS; Little Rock, AR, USA) where they were housed in standard caging on a 12 h light–dark schedule and received standard rodent chow low in soy (no. 2020X, Harlant Laboratories, Indianapolis, IN, USA) and water ad libitum until they were 6 months of age. At 6 months of age, the mice were shipped to the Brookhaven National Laboratory (BNL; Upton, NY, USA). After a one-week acclimation period, the mice were exposed to sham-irradiation, whole body ^16^O (600 MeV/n; 0.1, 0.25 and 1.0 Gy, dose rate: 0.25–0.26 Gy/min, *n* = 10/group), or whole body ^1^H (150 MeV; 0.5 and 1 Gy, *n* = 10/group). Dosimetry was performed by the NASA Space Radiation Laboratory physics dosimetry group at BNL to ensure the quality of exposure. For each exposure, unanesthetized animals were individually placed into clear Lucite boxes (3 × 1.5 × 1.5 in) with breathing holes. Sham-irradiated mice were placed into the same enclosures for the same amount of time, but were not exposed to radiation. One-day post-irradiation, the mice were returned to UAMS, where they were housed under conditions described above. 

For comparison, at 6 months of age, a cohort of male C57BL6J mice was exposed to γ-rays using a cesium−137 (137Cs) source (Mark 1 Model 68A, J L Shepherd & Associates, San Fernando, CA, USA) at UAMS, at a dose rate of 1 Gy/min and to total doses of 0.5 Gy, 1 Gy, or 3 Gy (*n* = 10/group). Unanesthetized mice were placed in a clear, well-ventilated pie-shaped Plexiglas holder with individual compartments and exposed in the 137Cs source irradiator on a turntable (6 rpm) for even radiation exposure. Radiation dosimetry was performed with DOSE-MAP^®^ gafchromic films (Ashland Specialty Ingredients, Wayne, NJ, USA) and with an ion chamber calibrated for cesium (Exradin A20, Standard Imaging, Middleton, WI, USA). For sham-irradiation, age-matched animals were placed in the pie-shaped holder for 5 min but not exposed to radiation. All procedures were approved by the Institutional Animal Care and Use Committee at UAMS (protocol numbers 3523 and 3754) and BNL (protocol number 477). 

### 4.2. Plasma Collection

At 14 and 90 days, separate cohorts of mice were sacrificed and blood were collected. For this purpose, mice were anesthetized with 3% isoflurane. A modified infusion set (27G with shortened tubing) was used to inject a single dose of heparin (30–40 U/kg) into the abdominal vena cava. The same infusion set was then used to draw a blood sample from the abdominal vena cava and collect in an EDTA-coated tube. The tube was immediately spun at 1000 RCF at 5 °C for 15 min, and plasma was collected in a regular Eppendorf tube and snap-frozen in liquid nitrogen. 

### 4.3. Plasma Metabolomics/Lipidomics Using UPLC-QToF

A total of 136 plasma samples from mice were analyzed for this study. Samples (5 µL) were prepared for mass spectrometric analysis using 195 µL extraction buffer (40% acetonitrile, 25% methanol and 35% water) containing internal standards. The samples were vortexed, incubated on ice for 15 min and centrifuged at 13,000 rpm for 20 min at 4 °C. The supernatant was transferred to another Eppendorf tube and 200 μL of 100% acetonitrile was added. After brief vortex and incubation on ice for 15 min, the samples were centrifuged at 13,000 rpm for 20 min at 4 °C. The supernatant was dried using speed vacuum. The dried samples were then reconstituted in 200 µL of 5% methanol, 1% acetonitrile and 94% water solution and transferred to mass spectrometry vials for acquisition on Waters- G2Qtof instrument. Each sample (2 µL) was injected to a Waters Acquity BEH C18 1.7 µm, 2.1 × 50 mm column using an Acquity UPLC system coupled with quadrupole time of flight mass spectrometer. The column temperature was set at 60 °C. The gradient mobile phase consisted of Solvent A— 100% water with 0.1% formic acid, Solvent B—100% acetonitrile with 0.1% formic acid and Solvent D—90% isopropanol and 10% acetonitrile with 0.1% formic acid. Each sample injection was run for 13 min at a flow rate for 500 µL/min. The gradient conditions started with 98% of Solvent A which decreased to 40% Solvent A at 4 min with a ramp of curve 6. At 8 min, the gradient changed to 2% Solvent A and 98% Solvent B. At 9.50 min, at a ramp of curve 6, the gradient conditions shifted to 98% Solvent D and 2% Solvent B and remained until 11 min before shifting to 50% of Solvent A and Solvent B for another 30 s at 12 min, the gradient changed to initial conditions of 98% Solvent A. The column eluent was then introduced by electrospray ionization in both positive and negative mode to quadrupole time of flight mass spectrometer (G2- QToF, Waters Corporation, Milford, MA, USA). The instrument was operated with a capillary voltage of 2.50 kV, and a cone voltage of 30 V. The source temperature was set at 120 °C and desolvation temperature at 500 °C. The cone gas flow was at 25 L/h and desolvation gas flow at 1000 L/h. Real-time mass correction was applied using a solution of leucine-enkephalin (0.1 ng/mL) [M+H]^+^ (*m/z* 556.2771), [M−H]^−^ (*m*/*z* 554.2615) in 500 mL 50:50 acetonitrile/water and 250 μL formic acid at an infusion rate of 10 μL/min utilizing the Waters Lockspray^®^ interface (Waters Corporation, Milford, MA, USA). Before and after samples were run, a mixture of six standards (acetaminophen: *m/z* 152.0712 [M+H]^+^/150.0555 [M−H]^−^, sulfaguanidine: *m/z* 215.0603 [M+ H]^+^/213.0446 [M−H]^−^, sulfadimethoxine: *m/z* 311.0814 [M+H]+/309.0658 [M−H]^−^, Val-Tyr-Val: *m/z* 380.2185 [M+H]^+^/378.2029 [M−H]^−^, terfenadine: *m/z* 472.3216 [M+H]^+^ and leucine-enkephalin: *m/z* 556.2771 [M+H]^+^/554.2615 [M−H]^−^) were run to ensure mass accuracy during batch acquisition (detailed in Supplementary Figure S1). A number of measures were used to ensure high quality and reproducibility of LC-MS data. For example, the column was conditioned using the pooled QC samples that were injected periodically (after every 10 sample injections) to monitor mass accuracy, shifts in retention time and signal intensities as measures of reproducibility and data quality of the LC-MS data. The overlap of QC sample chromatograms (base peak intensity) shows minimal shifts in retention time and consistency in peak intensities throughout the acquisition (detailed in Supplementary Figure S2). We also have blank runs interspersed between set of samples to minimize carry-over effects. We manually inspected for accurate peak detection/integration and performed principal component analysis to identify outliers. Replicate samples were included when the sample was in abundance. Finally, a mixture of metabolite standards was injected at the beginning and at the end of the acquisition batch so as to monitor mass accuracy below 5 ppm. 

### 4.4. Statistical Analyses of Mass Spectrometry Data

The raw data files obtained from the acquisition on the mass spectrometer were converted into NetCDF files for pre-processing. XCMS (an abbreviation for various forms (X) of chromatography mass spectrometry) [46] was used for pre-processing the data files and normalization was performed with internal standards in both positive and negative mode data. Metaboanalyst (v 3.0) was used to perform multivariate analysis. Statistically significant *m/z* with a *p*-value ≤ 0.05 were putatively identified with a database search by applying the online version of CEU Mass Mediator (CMM), which was integrated from METLIN, Human Metabolome Database (HMDB) and LIPID MAPS with a ppm error of less than 10. The MS/MS raw data files were converted to NetCDF (network common data form) files using Waters MassLynx Databridge Software, then further converted to MSP (microsoft patch) file format using our in-house R package. The MS/MS validation was then completed by the NIST 2017 MS/MS database and METLIN online database. Metabolite ID was verified by using tandem mass spectrometry-based fragmentation pattern matching with pure standards.

## 5. Conclusions

In summary, findings from this study highlight the importance of molecular phenotyping technologies for the development of biomarkers indicative of exposure to low dose high-LET radiation. Our results indicate that exposure to ^16^O radiation led to dose- and time-dependent alterations in plasma profiles, suggestive of dyslipidemia and an inflammatory phenotype which can result in long-term systemic changes. Moreover, ^16^O has a several-fold higher relative biological effectiveness in altering the plasma metabolome compared to ^1^H or γ-rays. Since space radiation has a complex LET spectrum, future studies aimed at investigating the effects of additional heavy ions and mixed ion fields are needed. In addition, the inclusion of female mice in these studies would help understand gender-specific metabolic responses to space-type exposures.

## Supplementary Materials

Supplementary materials can be found at https://www.mdpi.com/2218-1989/10/6/252/s1, Figure S1: Total ion chromatograms for standard mixture of compounds before (Panel A) and after (Panel B) to monitor mass accuracy over the batch acquisition, Figure S2: TIC (total ion chromatograms) overlays for pooled quality controls in electrospray positive (Panel A) and ESI (electrospray ionization) negative mode (Panel B) to monitor retention time drifts over time, Table S1: Tandem MS/MS validations for dysregulated metabolites in mice plasma, Table S2: List of dysregulated metabolites (ANOVA for all doses) after exposure to ^16^O radiation at days 14 and 90 post-irradiation, Table S3: List of dysregulated metabolites (ANOVA for all time points) after exposure to 0.1 Gy, 0.25 Gy or 1 Gy of 16O radiation, Table S4: List of dysregulated metabolites after exposure to ^16^O radiation at day 14 (ANOVA_post hoc comparison), Table S5: List of dysregulated metabolites after exposure to 0.5 Gy and 1 Gy of 1H and, 0.5 Gy, 1 Gy and 3 Gy of g radiation (ANOVA for all doses of radiation), Table S6: List of dysregulated metabolites after exposure to radiation at days 14 and 90 post-irradiation.
