# Supplementary material for: Identification of Plasma Lipidome Changes Associated with Low Dose Space-Type Radiation Exposure in a Murine Model
Source: Metabolites. 2020 Jun 17;10(6):252. doi: 10.3390/metabo10060252 (PMC7345467; doi:10.3390/metabo10060252)

Identification of plasma lipidome changes associated with low dose space-type radiation exposure in a murine model

[$]Maarisha Upadhyay[1], [$]Meena Rajagopal[1], Kirandeep Gill[1], Yaoxiang Li[1], Shivani Bansal[1], Vijayalakshmi Sridharan[2], John B Tyburski[1], Marjan Boerma[2] and Amrita K Cheema[1,3] *

[1]Department of Oncology, Lombardi Comprehensive Cancer Center, Georgetown University Medical Center, Washington, DC 20001, USA

[2]Division of Radiation Health, University of Arkansas for Medical Sciences, 4301 West Markham Slot 522-10, Little Rock, AR 72205, USA

[3]Department of Biochemistry, Molecular and Cellular Biology, Georgetown University Medical Center, Washington DC, USA

***** Correspondence: akc27@georgetown.edu; Tel.: 1-202-687-2756

$: Both authors contributed equally

Supplementary Figure S1. Total ion chromatograms for standard mixture of compounds before (Panel A) and after (Panel B) to monitor mass accuracy over the batch acquisition.

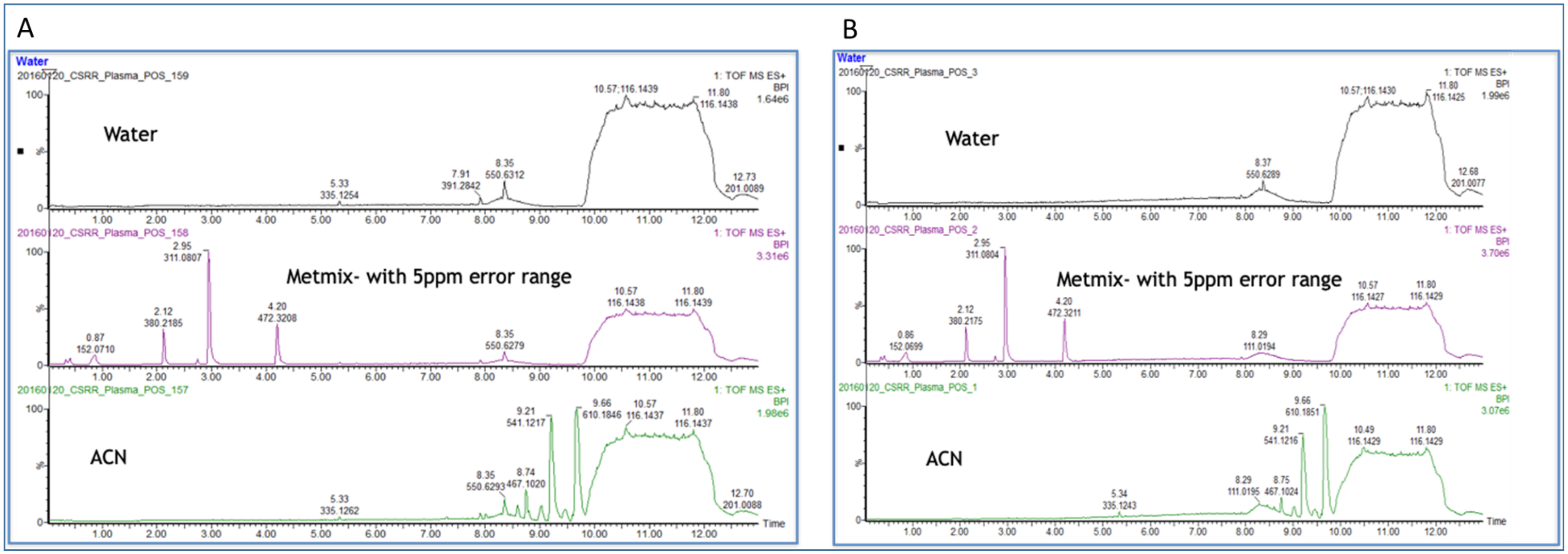

Supplementary Figure S2. TIC overlays for pooled quality controls in electrospray positive (Panel A) and ESI negative mode (Panel B) to monitor retention time drifts over time.

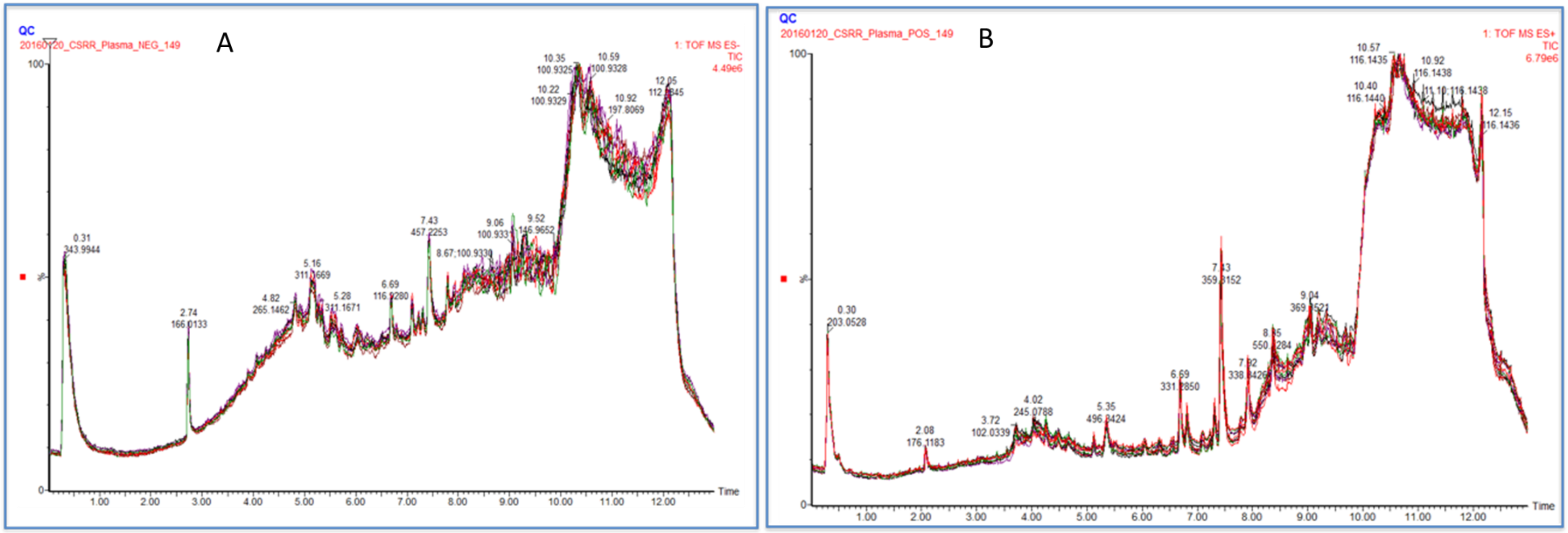

Supplement: Supplementary file 1 [file metabolites-10-00252-s001.zip › metabolites-817228-SI/metabolites-817228-supplementary-proofed/Supplementary File_Metabolites.pdf]
