# Supplementary figures and images for: Identification of Plasma Lipidome Changes Associated with Low Dose Space-Type Radiation Exposure in a Murine Model
Source: Metabolites. 2020 Jun 17;10(6):252. doi: 10.3390/metabo10060252 (PMC7345467; doi:10.3390/metabo10060252)

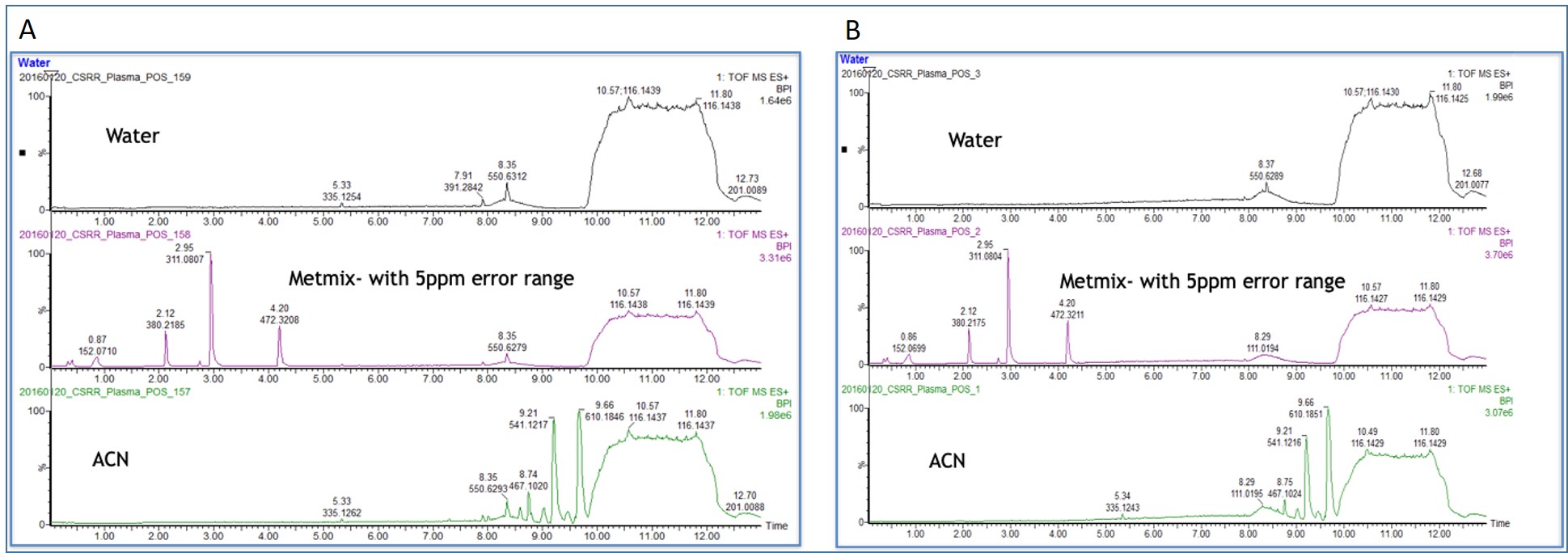

Supplement: Supplementary file 1 [file metabolites-10-00252-s001.zip › metabolites-817228-SI/metabolites-817228-supplementary-proofed/Supplementary Figure S1.jpg]

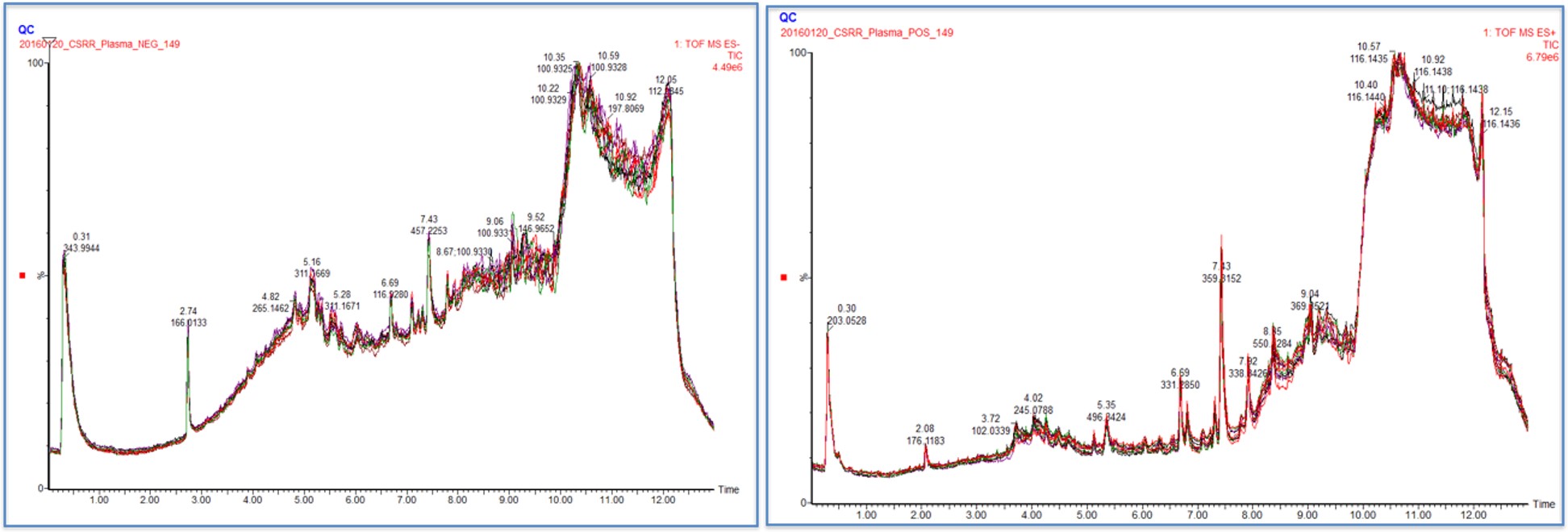

Supplement: Supplementary file 1 [file metabolites-10-00252-s001.zip › metabolites-817228-SI/metabolites-817228-supplementary-proofed/Supplementary Figure S2.jpg]
